# Root Silicon Addition Induces Fe Deficiency in Cucumber Plants, but Facilitates Their Recovery After Fe Resupply. A Comparison With Si Foliar Sprays

**DOI:** 10.3389/fpls.2020.580552

**Published:** 2020-12-10

**Authors:** Lourdes Hernández-Apaolaza, Laura Escribano, Ángel Mª Zamarreño, José Mª García-Mina, Carlos Cano, Sandra Carrasco-Gil

**Affiliations:** ^1^Department of Agricultural Chemistry and Food Science, Universidad Autónoma de Madrid, Madrid, Spain; ^2^Department of Environmental Biology, Sciences School, University of Navarra, Pamplona, Spain

**Keywords:** iron-deficiency, silicon, ROS, cellular cycle, phenolic compounds, phytohormones, cucumber

## Abstract

Silicon has not been cataloged as an essential element for higher plants. However, it has shown beneficial effects on many crops, especially under abiotic and biotic stresses. Silicon fertilization was evaluated for the first time on plants exposed to fluctuations in an Fe regime (Fe sufficiency followed by Fe deficiency and, in turn, by Fe resupply). Root and foliar Si applications were compared using cucumber plants that were hydroponically grown in a growth chamber under different Fe nutritional statuses and Si applied either to the roots or to the shoots. The SPAD index, Fe, and Mn concentration, ROS, total phenolic compounds, MDA concentration, phytohormone balance, and cell cycle were determined. The results obtained showed that the addition of Si to the roots induced an Fe shortage in plants grown under optimal or deficient Fe nutritional conditions, but this was not observed when Si was applied to the leaves. Plant recovery following Fe resupply was more effective in the Si-treated plants than in the untreated plants. A relationship between the ROS concentration, hormonal balance, and cell cycle under different Fe regimes and in the presence or absence of Si was also studied. The contribution of Si to this signaling pathway appears to be related more to the induction of Fe deficiency, than to any direct biochemical or metabolic processes. However, these roles could not be completely ruled out because several hormone differences could only be explained by the addition of Si.

## Introduction

Plants take up silicon in the form of monosilicic acid, Si(OH)_4_. Its concentration ranges in soil solutions from 0.1 to 1.4 mM (Marschner, 1995). Although this element has not been cataloged as essential for higher plants, the addition of Si to soils has exhibited beneficial effects for the growth of many plants, particularly under conditions of abiotic and biotic stresses (for a review, see Hernández-Apaolaza, 2014; Liang et al., 2015). The Si effect on the mitigation of different stresses has been studied in species such as cucumber (*Cucumis sativus*), rice (*Oryza sativa L*.), and soybean (*Glycine max*), among others (Miyake and Takahashi, 1985; Gonzalo et al., 2013; Carrasco-Gil et al., 2018). However, the mechanisms of action on the different stresses are not clear. Several authors have concluded that in plants subjected to multiple stresses, Si not only acts as a physical barrier in the plant cell but also is implicated in physiological activities (Liang et al., 2007; Pavlovic et al., 2013, 2016, etc.). Conversely, Coskun et al. (2019) proposed a model that concluded that the Si effects were indirectly caused by Si deposition in the apoplast and that there was no biochemical role for Si(OH)_4_ in terms of any interactions with intracellular constituents or enzymes.

Silicon fertilizers have been known worldwide since the middle of the eighteenth century, and as an alternative to root fertilization, foliar fertilization is a widely used and increasingly important crop nutrition strategy. The ability of leaves to absorb water and nutrients was recognized approximately three centuries ago and occurs on the leaf surface through the cuticle, cracks and imperfections, stomas, trichomes, and lenticels (Fernández et al., 2013). Foliar fertilization is considered to be more environmentally friendly when compared to root fertilization because the nutrients are supplied directly to the plant tissues during the critical stages of plant growth. However, it is noted that the efficacy of foliar fertilization can be much more uncertain when compared to root fertilization. In addition, foliar fertilizers may have a synergistic effect when applied together with fungicides or pesticides.

Iron (Fe) is an essential microelement for plants and other living organisms. It has multiple functions in plants, among which is its involvement in the synthesis of chlorophyll, and the development of thylakoids and chloroplasts is notable. However, even though the total content of Fe in calcareous soils is usually much higher than the needs of plants, its bioavailability is often very limited due to its precipitation as Fe(III) oxides and hydroxides (Guerinot and Yi, 1994). This effect causes an important nutritional disorder known as iron chlorosis responsible for the reduction in both yield and quality of a wide variety of crops. Therefore, higher plants have been forced to evolve different strategies to acquire Fe from the soil to meet their needs. The plants employ two different strategies to deal with Fe deficiency. Strategy I, which is used by dicotyledons and monocotyledons, but not grasses, consists of the development of biochemical changes such as the increase in Fe(III) reduction capacity mediated by the enzyme Fe reductase or the acidification of the rhizosphere by the excretion of protons through an H+ -ATPase pump. The plants that follow Strategy II acquire Fe by exuding phytosiderophores (Marschner and Römheld, 1994) and other chelating agents as phenolic compounds. For the first time, Bityutskii et al. (2010) observed that the application of Si to a nutrient solution could alleviate Fe deficiency symptoms in cucumber and pumpkin (*Cucurbita pepo*) (Strategy I plants), but not in maize (*Zea mays*) and barley (*Hordeum vulgare*) (Strategy II plants). This finding has been supported by other authors using Strategy I species such as cucumber (Pavlovic et al., 2013, 2016). Pavlovic et al. (2013) demonstrated that the application of Si in Fe-deficient cucumber plants increased the concentration of apoplastic Fe in roots and improved the expression of proteins involved in the absorption of Fe based on the Fe reduction processes. Research conducted by Pavlovic et al. (2016) showed that the supply of Si improved the relocation of Fe from older cucumber leaves to younger cucumber leaves and mitigated Fe deficiency in these plants by increasing the supply of this metal by apoplasts in the roots, and the accumulation of Fe mobilizing compounds in the roots (citrate and catechin) and leaves (citrate), which improved the acquisition and translocation of Fe to the apical parts of shoots (Pavlovic et al., 2013; Bityutskii et al., 2014). On the contrary, Gonzalo et al. (2013) observed that the addition of Si to cucumber plants had no effect on the alleviation of chlorosis symptoms in the leaves. They also observed that the response to Si addition under iron deficiency was plant-specific, and its beneficial effect on Fe deficient soybean was remarkable.

Carrasco-Gil et al. (2018) performed a combined approach including laser ablation-ICP coupled with mass spectrometer (LA-ICP-MS) images, to evaluate the effect of Si addition to Fe-deficient and sufficient rice plants. A different Si effect was observed depending on plant Fe status. Under Fe sufficiency, Si supply decreased Fe concentration inside the root and increased the oxidative stress in the plant, which is in accord with Coskun et al. (2019) model. Therefore, the Fe acquisition strategies were activated and the Fe translocation rate to the aerial part was increased, even under an optimal Fe supply. They hypothesized that this effect was related to an increase in Fe root plaque formation due to the presence of Si. For rice plants, highly stable iron-chelating agents like EDDHA or HBED could not be used, as they are stronger than the plant’s capacity to uptake the Fe from the chelate, so EDTA or inorganic salts were used to provide Fe to strategy II plants. Under Fe deficiency, the treated Si plants absorbed Fe from the plaque more rapidly than the non-treated Si plants, due to the previous activation of Fe deficiency strategies during the growing period (the plants were previously grown with both Fe and Si). Recently, Becker et al. (2020) reported a similar Fe uptake reduction and activation of Fe deficiency responses in rice due to the addition of Si to the growth media. These authors observed that Si reduced shoot Fe concentrations independently of the Fe concentration supplied or the Fe form tested (FeEDDHA, FeEDTA, and FeSO_4_). Similarly, Si addition decreased the apoplastic Fe concentrations in the roots. Furthermore, as a reaction to the Fe deficiency promoted by Si, the root Fe-homeostasis-related genes were upregulated, and a strategy to overcome the chlorosis was set up.

This work aimed to evaluate for the first time the effect of root and foliar Si application on cucumber plants grown at different Fe statuses: Fe sufficiency, Fe deficiency, and after re-fertilization with Fe to establish the utility of Si fertilizers for this crop. The mode of action of this beneficial element has been studied by the evaluation of ROS, phytohormones, and cell cycle behavior, under three Fe regimes and two types of Si application.

## Materials and Methods

### Plant Growth Conditions

Cucumber (*Cucumis sativus* L., cv. Ashley) seeds were sterilized with sodium hypochlorite (5%) for 5 min and were stored in distilled H_2_O for 24 h at 4°C. After that, two hundred seeds were germinated on filter-moistened paper with 1 mM CaSO_4_ for 72 h at 28°C. Seeds were moistened every 24 h with 1 mM CaSO_4_. Homogeneous seedlings (36 units) were transferred to 10-L plastic pots containing distilled H_2_O with continuous aeration for 4 days. Then, plants were transferred to 3-L opaque containers (6 plants per container) with diluted 1/5 Hoagland nutrient solution for 3 days more. The composition of the Hoagland nutrient solution was as follows: macronutrients (in mM) 1.0 Ca(NO_3_)_2_, 0.9 KNO_3_, 0.3 MgSO_4_, and 0.1 KH_2_PO_4_ and micronutrients (in μM): 35 NaCl, 10 H_3_BO_3_, 0.05 Na_2_MoO_4_, 2.5 MnSO_4_, 1.0 CuSO_4_, 10 ZnSO_4_, 1.0 CoSO_4_, 1.0 NiCl_2_, and 115.5 EDTANa_2_ (to avoid metal precipitation in the nutrient solution). Iron was added when required as 20 μM Fe-HBED (N,N’-bis(2-hydroxy benzyl)ethylenediamine-N,N’-diacetic). The pH of the nutrient solution was adjusted to pH 7.5 ± 0.1 by the addition of 3 mM HEPES and readjusted every day with 1.0 M KOH or 1.0 M HNO_3_. Besides, 0.3 g of CaCO_3_ per pot was introduced to simulate calcareous soil conditions and increase buffer capacity. Then, plants were submitted to a complete Hoagland solution and three different Si treatments were imposed: Si addition to roots (+SiR), Si sprayed to leaves (+SiF), and no Si addition (-Si). For the +SiR treatment, 1.5 mM Si(OH)_4_ was supplied to the roots by adding it to the nutrient solution, which was renewed every week. For the +SiF plants, 1.5 mM Si(OH)_4_ solution at pH 5.0, to avoid altering the ion exchange properties of the cuticle, was sprayed to each leave of the plants treated [three doses (125 μL each) per leave per week]. The foliar spray covered the full leaf, and no extra formulation of the foliar solution was needed as it stayed on the leaf without dripping. Along the experiment, plants presented similar number of leaves for the three Si treatments tested. Finally, a control without Si was performed (-Si). Two sets of three pots for each Si treatment were available (6 pots for +SiR, 6 for +SiF, and 6 for –Si). As mentioned before, each pot has six plants.

After 7 days of growth, plants of each Si treatment were divided into two, in half of the plants (three pots with six plants each) Fe was removed from the nutrient solution (-Fe), and in the other half Fe supplied was maintained. Finally, after 7 days more, Fe resupply to the deficient plants were carried out. To differentiate the capacity of the plants to take up Fe from this resupply, the Fe re-fertilization phase has been done with 20 μM ^57^Fe-HBED (+FeR) for 5 days. Plants were grown in a growth chamber with a photosynthetic photon flux density at leaf height of 350 μmol m^–2^ s^–1^ photosynthetically active radiation, 16 h, 25°C, 40% humidity (day) and 8 h, 20°C, 60% humidity (night). A total of three biological replicates per treatment (1 pot each) were performed.

For the preparation of Fe-HBED, HBED was dissolved in KOH (molar ratio 1:3). Then, Fe(NO_3_)_3_ (chelating agent: Fe molar ratio of 1:1) was added to this solution, using 2% excess Fe to ensure that all the chelating agent was complexed by Fe. The pH range during the complexing process was controlled to be 5.0–8.0. Finally, the pH of the chelate solution was adjusted to pH 7 and made up to volume with MilliQ water. After 24 h, it was filtered to eliminate the Fe precipitated in the form of oxides. Silicic acid was prepared by passing a solution of 0.5 M Na_2_SiO_3_ through a column filled with cation exchange resin (Amberlite IR-120, Na+ form; Fluka, Buchs SG, Switzerland) (Nikolic et al., 2007). The preparation of the isotopically marked Fe chelate was as follows: ^57^Fe_2_O_3_ (with 96.66% isotopic enrichment; Isoflex, San Francisco, CA, United States) was dissolved in the minimum amount of concentrated HCl, forming ^57^FeCl_3_ (suprapur; Merck, Darmstadt, Germany). Finally, ^57^FeCl_3_ was slowly added to the chelating agent dissolved in KOH in the same way as indicated above, maintaining the restrictions of pH intervals (5–8).

### Plant Analysis

The degree of chlorosis of the leaves was quantified by a non-destructive method using the SPAD (Soil and Plant Analyzer Development) model 502 (Minolta Co., Osaka, Japan) digital chlorophyll meter.

Plant material was sampled at the end of each period of growth: Fe sufficient (+Fe), Fe deficient (-Fe) conditions, and also at the end of the re-fertilization phase (+FeR). Plant material was divided into root, stem, old leaves (grown before Fe deficiency conditions), and new leaves (grown after Fe deficiency). Half of the plant material was frozen in liquid N_2_ and stored at -80°C for analysis of the stress indexes. The rest of the plant material was washed with 0.1% Tween 80 (v/v) and 1% HCl (v/v) and rinsed twice with distilled water. Then, plant tissues were put in an oven at 60°C for 72 h until constant weight and the dry weight (DW) was determined. Iron and manganese concentration was quantified after microwave (CEM Corporation MARS 240/50; Matthews, NC, United States) digestion with HNO_3_ 65% and H_2_O_2_ 30% by atomic absorption spectrophotometry (AAs) (PerkinElmer Analyst 800. The ^57^Fe concentration was also determined after the resupply phase, by ICP-MS (7500 c, Agilent Technologies, California, United States), and its quantification was carried out by isotope pattern deconvolution analysis (Rodriguez-Castrillon et al., 2008).

The total phenolic concentration was determined using Folin–Ciocalteu reagent following the procedure described by Ainsworth and Gillespie (2007). The fresh plant material (0.15 g) was placed together with 3 mL 95% MeOH in mortar. The homogenate was incubated for 48 h at room temperature in the dark and centrifuged 15 min at 9,000 rpm and 25°C. After that, 100 μL of the supernatant was mixed with 200 μL of 10% Folin–Ciocalteu reagent and 800 μL of 700 mM Na_2_CO_3_, incubated 40 min at 40°C and centrifuged 15 min at 14,000 rpm and 20°C. The resulting chromophore was measured at 765 nm in a UV-Visible spectrophotometer (SPECTROstar Nano, BMG LABTECH, Germany). Gallic acid was used as a standard compound, and the total phenols were expressed as mmol gallic acid/g fresh weight.

For reactive oxygen species (ROS) analysis, fresh plant material (0.2 g) was chopped in 2 mL 50 mM HEPES at pH 7. The extract (50 μL) was mixed with 150 μL 50 mM HEPES and 4 μL 5 μM H_2_DCFDA (diacetate of 2’,7’-diclorodihydrofluorescein) (Molecular Probes, Invitrogen, Carlsbad, CA, United States) and incubated 30 min at 37°C in agitation (100 rpm). Then, the extract was centrifuged at 1,000 rpm for 10 min and the pellet was resuspended in 0.2 ml HEPES and incubated for 10 min more at 37°C. The fluorescence intensity of DCF was measured with a fluorescence spectrophotometer (Cary Eclipse Fluorescence, Varian, Australia) at room temperature, with an excitation wavelength of 488 nm and emission filter between 500 and 600 nm (the excitation and emission slits width 5 nm). The fluorescence intensity was used to determine relative ROS production.

For the analysis of lipid peroxidation of plant cell membranes, the method of Buege and Aust (1978) was followed, for which the thiobarbituric reagent TCA–TBA–HCl was prepared with a composition of trichloroacetic acid (TCA) 15%, thiobarbituric (TBA) 0.37%, HCl 0.25 M, and butylhydroxytoluene (BHT) 100 mg/L. The fresh plant material was placed together with the thiobarbituric reagent in a mortar. The homogenate was incubated for 30 min at 90°C and centrifuged at 14,000 rpm. The supernatant was analyzed by measuring absorbance at 535 and 600 nm in a double-beam UV-Visible spectrophotometer (Jasco V-650). All the reagents were from Sigma Aldrich. The quality of the reagents used was for TCA: ACS reagent, ≥99.0%, for TBA: ≥98%, and for BHT: analytical standard of malondialdehyde (MDA).

For cellular cycle analysis, fresh plant material (0.3 g) was chopped with 1 mL of Otto I reagent [0.1 M citric acid, 0.5% Tween 80 (v/v)] in a Petri dish. The homogenate was filtered (45 μm) and mixed with 2 mL Otto II (0.4 M Na_2_HPO_4_), propidium iodide, and RNAase were added to the extract to achieve the final concentration of 50 μg/ml for both reagents (Dolezel et al., 2007). The analysis was performed by flow cytometry (Cytometer BD FACSCanto II, Biosciences, United States).

The concentration in leaves of indole-3-acetic acid (IAA), abscisic acid (ABA), salicylic acid (SA), jasmonic acid (JA), and JA-Ile was determined by using high-performance liquid chromatography–electrospray–high-resolution orbitrap mass spectrometry (HPLC–ESI–HRMS). The extraction, purification, and quantification of these plant hormones were carried out as described in Silva-Navas et al. (2019). In brief, 0.25 g of ground frozen plant tissue was taken and homogenized with 2.5 mL of the mixture of methanol: water: HCOOH (90:9:1, v/v/v) and 2.5 mM Na-diethyldithiocarbamate) precooled at −20°C. Also, an internal standard of deuterium-labeled in methanol was added (25 μL of a stock solution of 1,000 ng ml^–1^). Samples were shaken for 60 min at 2,000 rpm at room temperature in a Multi Reax shaker and centrifuged at 20,000 g for 10 min. A re-extraction was carried out with 1.25 mL of fresh extraction mixture by shaking for 20 min and centrifugation. After this, 2 mL of the pooled supernatants was rota-evaporated at 40°C in a RapidVap Evaporator, and 500 μL of methanol/0.133% acetic acid (40:60, v/v) was used to resuspend the residue. Finally, it was centrifuged at 20,000 g for 10 min and injected in the HPLC–ESI–HRMS system. A detailed description of the quantification is reported in Silva-Navas et al. (2019). Similarly, the concentration in plant organs of several cytokinins was analyzed [trans- and cis-zeatin (tZ and cZ), dihydrozeatin (DHZ), trans- and cis-zeatin riboside (tZR and cZR), dihydrozeatin riboside (DHZR), isopentenyladenine (iP), and isopentenyladenosine (iPR)] by using HPLC–ESI–HRMS. For that purpose, the extraction and purification of these plant hormones were carried out as described in Silva-Navas et al. (2019). In this case, 0.25 g of plant material previously ground with liquid nitrogen was homogenized with 4 mL of methanol–water–formic acid (15:4:1, v/v/v) solution precooled at −20°C. As for the previous hormones, a deuterium-labeled standard in methanol was used (25 μL of a stock solution of 1,000 ng/mL). Solids were separated after 8 h extraction at −20°C followed by its centrifugation (20,000 g, 10 min, 4°C), and the same was repeated with 2 mL of extraction mixture. Supernatants were purified by passing them through a Sep-Pak C18 cartridge which was preconditioned with 2 mL of methanol and 2 mL of extraction medium. Then, the liquid was kept near to dryness in a RapidVap Evaporator and the residue was re-dissolved in 2 mL of 1 M formic acid, which was passed through an Oasis MCX column, where cytokinin bases and ribosides were eluted with 2 mL of NH_4_OH 0.35 M, in methanol 60% (v/v). The solvent of the obtained solution was again evaporated, and the sample redissolved in 250 μL of methanol +250 μL of 0.04% formic acid and finally centrifuged at 20,000 g for 10 min, before its injection in HPLC–ESI–HRMS. The quantitation method and data processing were carried out as described in Silva-Navas et al. (2019). All reagents employed were of ultrapure grade.

### Statistical Analysis

Statistical analysis was carried out with SPSS for Windows (v. 26.0), using unidirectional variance analysis (ANOVA). The comparisons of means were carried out using Duncan’s test (*p* < 0.05).

## Results

Data obtained will be presented according to the Fe nutritional status of the plant. Therefore, the results of Fe sufficient, Fe deficient, and Fe resupply plants will be shown separately.

### Effect of Si Addition on Fe Sufficient Cucumber Plants

**FIGURE 1:**
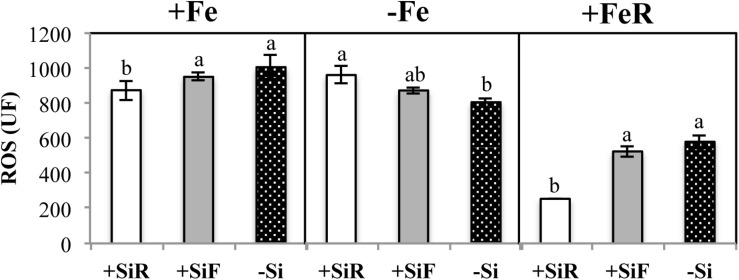
ROS reactive oxygen species (UF) in new leaf of cucumber plants under different Fe treatments [21 days of Fe sufficiency (+Fe), 10 days of sufficiency + 7 days of Fe deficiency (−Fe) and 5 days more of refertilization with ^57^Fe (+FeR)] and Si treatments [Si applied to root (+SiR), leaves (+SiF), and no Si (−Si) application]. The data are the mean ± SE (*n* = 3). Different letters in the same row showed significant differences according to Duncan’s test (*p* < 0.05).

**FIGURE 2:**
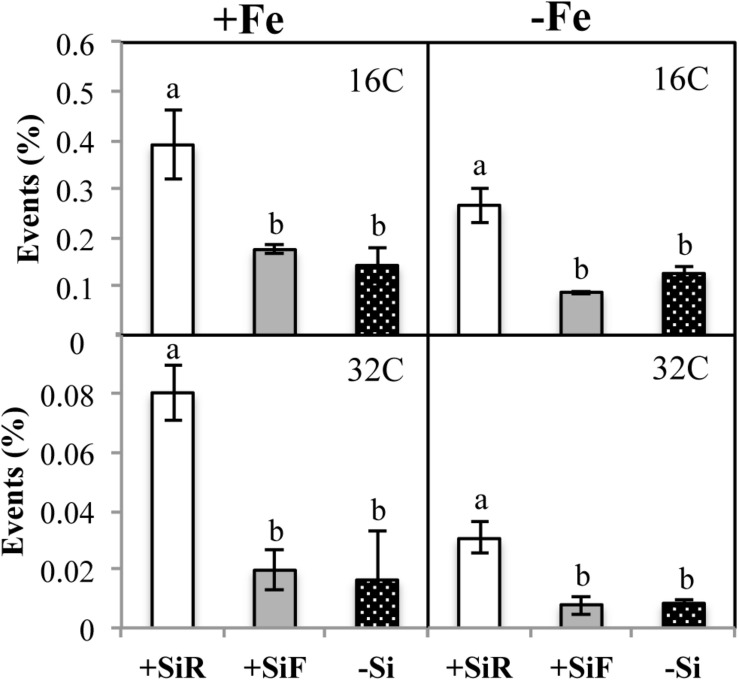
Cellular cycle (% events) in new leaf of cucumber plants under different Fe [21 days of Fe sufficiency (+Fe) and 10 days of sufficiency + 7 days of Fe deficiency (−Fe)] and Si [Si applied to root (+SiR), leaves (+SiF), and no Si (−Si) application] treatments. The data are the mean ± SE (*n* = 3). Different letters in the same row showed significant differences according to Duncan’s test (*p* < 0.05).

The SPAD index of new leaves under Fe sufficiency (+Fe) decreased significantly when Si was added to the nutrient solution (+SiR treatment) compared with the control plants without Si addition (–Si). However, Si application to leaves did not alter the SPAD index (Table 1). No differences were found in the dry weight (DW) as a result of the Si treatment used (+SiR, +SiF, -Si) (Table 1). The Fe concentration in the old leaves of the +SiR plants was significantly lower than in the untreated Si plants (-Si), but no differences were observed when foliar Si was added (+SiF) (Table 2). When the Fe concentration of the new leaves was analyzed, no differences were obtained between the Si-treated and the untreated plants (Table 2). A significantly lower Fe/Mn ratio was found in the old and the new leaves of the +SiR plants, but when Si was sprayed onto the leaves, a decreased Fe/Mn ratio was only observed in the old leaves (Table 2). No differences in the Fe/Mn ratio in the roots were observed related to the Si supply. Furthermore, +SiR significantly reduced ROS production (Figure 1) and increased the phenolic compound concentration in the new leaves (Table 3) when compared with the –Si plants. When Si was applied to the leaves (+SiF), the ROS concentration and total phenol concentration remained similar to the untreated plants. The malondialdehyde (MDA) concentration data indicated that no cell damage was detected in any of the treatments (Table 3). The cellular cycle did not show any significant differences in ploidy levels 1C, 2C, 4C, and 8C (data not shown) after the Si treatments. However, the ploidy levels 16C and 32C (Figure 2), significantly increased in the +SiR application when compared with the +SiF and –Si treatments, which showed a lower grade of cellular division and, therefore, a greater cellular size.

**TABLE 1:** SPAD index and dry weight (DW, g) in new leaves of cucumber plants under different Fe treatments [21 days Fe sufficiency (+Fe), 10 days of sufficiency + 7 days of Fe deficiency (−Fe) and 5 days more of refertilization with ^57^Fe ((−Fe) + Fe)] and Si treatments [Si applied to root (+SiR), leaves (+SiF), and no Si (−Si) application].

	**+SiR**	**+SiF**	**(−Si)**

	**SPAD index**		
**+Fe**	40.4 ± 0.9*b*	45.9 ± 1.1*a*	45.4 ± 0.8*a*
**(−Fe)**	26.6 ± 0.6*a*	27.1 ± 1.0*a*	25.8 ± 0.9*a*
**(−Fe)+Fe**	30.9 ± 0.8*a*	32.4 ± 0.9*a*	25.6 ± 1.4*b*

**DW (g)**			

**+Fe**	0.17 ± 0.03*a*	0.10 ± 0.06*a*	0.14 ± 0.05*a*
**(−Fe)**	0.17 ± 0.05*a*	0.15 ± 0.06*a*	0.10 ± 0.02*a*
**(−Fe)+Fe**	0.36 ± 0.13*a*	0.34 ± 0.10*a*	0.36 ± 0.07*a*

*The data are the mean ± SE (n = 3). Different letters in the same row showed significant differences according to Duncan’s test (p < 0.05).*

**TABLE 2:** Iron concentration (μg/g DW) in old and new leaves and Fe/Mn ratio in roots and old and new leaves of cucumber plants under different Fe treatments [21 days Fe sufficiency (+Fe), 10 days of sufficiency + 7 days of Fe deficiency (−Fe), and 5 days more of refertilization with ^57^Fe ((−Fe) + Fe)] and Si treatments [Si applied to root (+SiR), leaves (+SiF), and no Si (−Si) application].

	**+SiR**	**+SiF**	**(−Si)**
	**Fe concentration(μ g g-^1^)**		

	Old leaves		

**+Fe**	88.9 ± 2.9*b*	99.8 ± 5.6*ab*	104.1 ± 1.1*a*
**(−Fe)**	42.8 ± 3.7*b*	64.5 ± 6.7*a*	65.4 ± 3.6*a*
**(−Fe)+Fe**	103.0 ± 6.3*b*	151.6 ± 9.0*a*	110.0 ± 8.10*b*

	New leaves		

**+Fe**	109.9 ± 12.7*b*	167.8 ± 7.8*a*	121.9 ± 9.2*ab*
**(−Fe)**	41.4 ± 13.4*b*	60.8 ± 3.1*ab*	80.9 ± 13.0*a*
**(−Fe)+Fe**	140.3 ± 12.2*a*	113.8 ± 5.9*a*	134.2 ± 7.0*a*

	**Fe/Mn**		

	Old leaves		

**+Fe**	0.653 ± 0.113*b*	0.569 ± 0.040*b*	0.798 ± 0.085*a*
**(−Fe)**	0.419 ± 0.037*b*	0.650 ± 0.199*a*	0.698 ± 0.146*a*
**(−Fe) +Fe**	0.853 ± 0.064*a*	1.083 ± 0.045*a*	0.970 ± 0.274*a*

	New leaves		

**+Fe**	0.900 ± 0.080*b*	1.157 ± 0.186*a*	1.260 ± 0.017*a*
**(−Fe)**	0.637 ± 0.162*b*	0.653 ± 0.137*b*	1.144 ± 0.091*a*
**(−Fe)+Fe**	1.573 ± 0.170*a*	1.120 ± 0.617*a*	1.247 ± 0.032*a*

	Root		

**+Fe**	0.997 ± 0.081*b*	1.367 ± 0.251*a*	1.125 ± 0.232*ab*
**(−Fe)**	1.646 ± 0.039*a*	1.266 ± 0.210*b*	1.540 ± 0.109*a*
**(−Fe)+Fe**	1.077 ± 0.025*a*	1.033 ± 0.078*a*	0.960 ± 0.080*a*

*The data are the mean ± SE (n = 3). Different letters in the same row showed significant differences according to Duncan’s test (p < 0.05).*

**TABLE 3:** Total phenol concentration (mmol gallic acid/g FW) and MDA (nmol/g FW) in leaves of cucumber plants under different Fe treatments [21 days Fe sufficiency (+Fe), 10 days of sufficiency + 7 days of Fe deficiency ((−Fe)), and 5 days more of refertilization with ^57^Fe ((−Fe) + Fe)] and Si treatments [Si applied to root (+SiR), leaves (+SiF), and no Si (−Si) application].

	**+SiR**	**+SiF**	**(−Si)**
	**Total phenols (mmol galic acid/g FW)**		

	Old leaves		

**+Fe**	12.9 ± 6.2*ab*	13.4 ± 4.0*ab*	17.4 ± 6.7*a*
**(−Fe)**	36.7 ± 6.2*a*	31.9 ± 6.0*ab*	29.1 ± 7.0*b*
**(−Fe)+Fe**	20.8 ± 4.7*b*	25.1 ± 3.2*a*	19.5 ± 2.3*b*

	New leaves		

**+Fe**	34.0 ± 4.9*a*	27.8 ± 5.5*b*	28.5 ± 3.1*b*
**(−Fe)**	30.0 ± 4.0*a*	39.4 ± 8.9*a*	34.7 ± 8.7*a*
**(−Fe)+Fe**	22.9 ± 4.2*a*	21.0 ± 3.6*a*	16.8 ± 3.5*a*

	**MDA (nmol/g FW)**		

	Old leaves		

**+Fe**	19.58 ± 6.37*a*	21.59 ± 1.95*a*	16.30 ± 3.79*a*
**(−Fe)**	7.80 ± 0.25*b*	12.74 ± 2.95*a*	8.91 ± 3.31*ab*
**(−Fe)+Fe**	19.01 ± 3.52*a*	19.36 ± 2.73*a*	11.20 ± 2.09*a*

	New leaves		

**+Fe**	18.68 ± 1.94*a*	17.62 ± 2.31*a*	12.47 ± 3.64*a*
**(−Fe)**	9.97 ± 3.46*a*	5.56 ± 1.23*b*	5.99 ± 0.69*b*
**(−Fe)+Fe**	15.08 ± 2.04*a*	13.12 ± 1.97*a*	9.15 ± 2.11*b*

*The data are the mean ± SE (n = 3). Different letters in the same row showed significant differences according to Duncan’s test (p < 0.05).*

The phytohormone concentration was also assessed in this study (Table 5). The auxin, indoleacetic acid (IAA) significantly increased in the roots of either the +SiR or +SiF treatments. Considering the old leaves, no differences were observed with or without Si application. However, in the new leaves, the +SiR treatment increased the IAA concentration and the concentration of +SiF was reduced with respect to -Si. The abscisic acid concentration in the roots increased only in the +SiF treatment compared with –Si but significantly decreased in the old leaves of the plants +SiR and +SiF. No differences in ABA concentration in the new leaves were detected. Likewise, no differences in cytokinins (CKs) in either the root or the old leaves were obtained, but in the new leaves, both Si applications increased the CK concentration, especially in the foliar supply of Si. No consistent differences were obtained in the salicylic acid (SA) concentration among the Si applications. Both types of Si application (+SiR and +SiF) decreased JA-Ille in the roots and the old leaves, but in the new leaves only +SiF decreased this hormone value. The jasmonic acid data showed that this hormone decreased in the roots and the old leaves only after the +SiF supply, but this effect was not detected in the new leaves (Table 5).

**TABLE 4:** SPAD increment (SPAD index after Fe resupply-SPAD index before Fe resupply) and ^57^Fe content (μg/organ) of the old leaves at the 3-day resupply stage under different Si treatments [Si applied to root (+SiR), leaves (+SiF), and no Si (−Si) application].

	**+SiR**	**+SiF**	**(−Si)**

	**SPAD increment after Fe resupply**		
**+Fe**	1.7 ± 0.2*b*	5.5 ± 1.5*a*	8.1 ± 2.0*a*
**(−Fe)+Fe**	6.5 ± 1.6*b*	11.7 ± 3.0*a*	0.7 ± 0.2*c*

	**^57^Fe content in old leaves (μg/organ)**		

**(−Fe)+Fe**	11.7 ± 4.2*ab*	19.6 ± 4.37*a*	8.6 ± 3.30*b*

*The data are the mean ± SE (n = 3). Different letters in the same column showed significant differences according to Duncan’s test (p < 0.05).*

**TABLE 5:** Phytohormone concentration (pmol/g PF) of abscisic acid (ABA), salicylic acid (SA), indoleacetic acid (IAA), jasmonic acid (JA), jasmonyl-L-isoleucine (JA-Ile), and cytokinins) in new leaf of cucumber plants under different Fe treatments [21 days of Fe sufficiency (+Fe), 10 days of sufficiency + 7 days of Fe deficiency ((−Fe)), and 3 days more of refertilization with ^57^Fe ((−Fe) + Fe)] and Si treatments [Si applied to root (+SiR), leaves (+SiF), and no Si ((−Si)) application].

**Phytohormones Concentration (pmol / g _*FW*_)**										
		**+Fe**			**(−Fe)**			**(−Fe)+Fe**		

		**+SiR**	**+SiF**	**(−Si)**	**+SiR**	**+SiF**	**(−Si)**	**+SiR**	**+SiF**	**(−Si)**

**Root**	**ABA**	3.28 ± 0.57*b*	4.59 ± 0.84*a*	2.69 ± 0.77*b*	2.2 ± 1.0*b*	4.1 ± 0.4*a*	2.6 ± 0.2*b*	3.14 ± 0.14*b*	4.53 ± 0.75*a*	3.63 ± 0.39*b*
	**SA**	484.7 ± 259.0*b*	684.1 ± 116.2*a*	652.3 ± 64.2*ab*	635.4 ± 166.8*b*	457.4 ± 23.7*b*	970.8 ± 175.6*a*	554.3 ± 50.1*b*	1190.3 ± 476.5*a*	1565.2 ± 255.1*a*
	**IAA**	43.4 ± 3.3*a*	47.3 ± 5.3*a*	35.3 ± 5.3*b*	38.0 ± 12.1*a*	38.7 ± 5.5*a*	12.5 ± 1.4*b*	28.4 ± 5.9*b*	48.1 ± 4.1*a*	30.3 ± 8.2*b*
	**JA**	30.0 ± 0.5*a*	19.6 ± 3.5*b*	39.6 ± 15.3*a*	19.2 ± 2.2*a*	9.3 ± 0.3*b*	7.2 ± 2.4*b*	20.8 ± 8.4*a*	17.8 ± 2.9*a*	10.7 ± 0.3*b*
	**JA- Ile**	0.55 ± 0.08*b*	0.55 ± 0.11*b*	1.58 ± 0.19*a*	1.13 ± 0.19*a*	0.78 ± 0.26*a*	0.98 ± 0.46*a*	1.05 ± 0.03*a*	0.38 ± 0.09*c*	0.50 ± 0.10*b*
	**Ck**	3.94 ± 0.96*a*	3.58 ± 0.10*a*	4.49 ± 0.48*a*	3.31 ± 0.10*b*	3.66 ± 0.10*ab*	3.99 ± 0.58*a*	5.18 ± 0.34*a*	3.93 ± 0.26*b*	4.14 ± 0.79*b*
**Old Leaves**	**ABA**	68.7 ± 5.4*b*	65.9 ± 3.3*b*	77.4 ± 9.5*a*	53.3 ± 14.9*a*	42.4 ± 17.4*a*	46.8 ± 10.1*a*	114.9 ± 28.1*a*	45.6 ± 4.3*b*	51.8 ± 3.0*b*
	**SA**	477.1 ± 0.3*b*	724.7 ± 84.9*a*	404.8 ± 74.5*b*	553.2 ± 8.0*b*	962.1 ± 44.5*b*	2011.6 ± 845.4*a*)	680.4 ± 229.5*ab*	546.8 ± 127.9*b*	756.2 ± 75.7*a*
	**IAA**	8.9 ± 0.7*a*	10.9 ± 1.4*a*	10.0 ± 2.6*a*	6.3 ± 1.0*b*	11.5 ± 3.1*a*	9.4 ± 1.5*a*	11.7 ± 1.2*a*	12.5 ± 2.4*a*	11.5 ± 0.3*a*
	**JA**	22.9 ± 4.2*a*	15.6 ± 0.9*b*	25.6 ± 5.8*a*	32.2 ± 3.1*a*	22.0 ± 7.1*b*	27.3 ± 2.7*ab*	93.9 ± 14.3*b*	21.7 ± 7.3*c*	183.2 ± 98.2*a*
	**JA- Ile**	1.07 ± 0.05*b*	0.38 ± 0.12*c*	2.01 ± 0.48*a*	1.55 ± 0.54*b*	3.74 ± 0.37*a*	0.69 ± 0.06*c*	5.58 ± 2.17*b*	3.72 ± 2.31*b*	13.4 ± 5.66*a*
	**Ck**	3.93 ± 0.47*a*	3.48 ± 0.36*a*	3.72 ± 0.68*a*	3.37 ± 0.10*a*	3.43 ± 0.28*a*	2.78 ± 0.45*b*	3.84 ± 0.15*b*	5.26 ± 0.32*a*	4.75 ± 0.21*b*
**New Leaves**	**ABA**	56.4 ± 15.9*a*	42.0 ± 9.1*a*	54.5 ± 9.7*a*	87.4 ± 2.3*a*	38.8 ± 9.0*b*	38.8 ± 13.1*b*	83.5 ± 16.9*a*	45.6 ± 9.3*b*	57.8 ± 12.4*b*
	**SA**	503.6 ± 23.5*a*	389.9 ± 77.9*b*	474.9 ± 61.2*a*	1305.0 ± 66.9*b*	2418.1 ± 571.8*a*	743.4 ± 11.3*c*	802.6 ± 121.1*a*	590.5 ± 267.5*a*	748.1 ± 173.7*a*
	**IAA**	12.7 ± 2.9*a*	8.5 ± 0.5*c*	11.1 ± 0.9*b*	13.7 ± 1.7*a*	5.0 ± 0.5*c*	10.1 ± 1.3*b*	14.3 ± 0.9*a*	16.7 ± 0.1*a*	16.7 ± 4.4*a*
	**JA**	44.5 ± 15.8*a*	30.2 ± 3.8*a*	47.0 ± 13.4*a*	83.4 ± 1.7*a*	24.7 ± 6.0*c*	57.8 ± 11.0*b*	138.9 ± 3.5*a*	43.1 ± 3.1*b*	45.6 ± 9.9*b*
	**JA- Ile**	1.62 ± 0.29*a*	0.84 ± 0.12*b*	1.98 ± 0.74*a*	2.40 ± 0.63*a*	0.87 ± 0.28*b*	0.98 ± 0.45*b*	7.6 ± 3.40*a*	0.95 ± 0.11*c*	3.91 ± 0.39*b*
	**Ck**	4.01 ± 0.66*b*	5.59 ± 0.36*a*	3.29 ± 0.26*c*	2.88 ± 0.05*a*	1.99 ± 0.36*b*	1.96 ± 0.13*b*	3.93 ± 0.37*a*	4.47 ± 0.21*a*	4.38 ± 0.57*a*

*The data are the mean ± SE (n = 3). Different letters in the same row showed significant differences according to Duncan’s test (p < 0.10).*

### Effect of Si Addition on Fe-Deficient Cucumber Plants

No significant differences in the SPAD index and the dry weight of the new leaves were found in the plants grown with or without Si (Table 1). For the +SiR treatment, a significant decrease in Fe concentration and Fe/Mn ratio in the old and new leaves compared with the –Si plants was observed, but no differences in Fe/Mn in the roots were found (Table 2). In the case of +SiF, no differences in the Fe concentration in the old and new leaves were observed between this treatment and the -Si plants (Table 2), nor was there any difference in the Fe/Mn ratio in the old leaves. In the new leaves and roots, the +SiF supply significantly decreased the Fe/Mn ratio. The OS concentration of the +SiR-treated plants was significantly higher than that of –Si, but no differences were detected between +SiF and -Si (Figure 1). The total phenol concentration (Table 3) was significantly increased in the old leaves of the +SiR when compared to the -Si plants, but no significant differences were detected in the new leaves. In the old leaves, the cell damage (Table 3) was similar for both Si applications when compared with the plants without Si supply, but in the new leaves, an increase in the MDA concentration was observed with the +SiR treatment. The 16C and 32C ploidy levels were significantly higher in the +SiR plants (Figure 2).

The hormone responses to Fe deficiency were also determined under Fe deficiency conditions (Table 5). The IAA in the roots significantly increased in both treatments (+SiR and +SiF) but decreased in the old leaves for +SiR compared with the Si-untreated plants. In the case of the new leaves, the IAA decreased for +SiF and increased for +SiR. The ABA concentration in the roots increased for the +SiF treatment when compared with –Si, and the new leaves exhibited a greater increase in the +SiR plants. Cytokinins (CKs) were reduced in the roots of the +SiR plants. However, in the old leaves a significant increase in both +SiR and +SiF was found, as well as in the new leaves of +SiR. The SA concentration in the root and old leaves decreased in the +SiR and +SiF treatments when compared with the plants without Si addition. However, an increase in the SA concentration in the new leaves treated with +SiR and +SiF was found especially in the +SiF treatment. The JA concentration in the roots and new leaves of the +SiR plants significantly increased in the +SiR treatment when compared to the +SiF- and –Si-treated plants. However, the foliar treatment with Si decreased the JA concentration in the new leaves. The JA-ille concentration in the old leaves increased in +SiR and +SiF, especially in +SiF, and in the new leaves of +SiR plants.

### Effect of Si Addition After Fe Resupply of Cucumber Plants

During the Fe resupply (–Fe + Fe), the plants treated either with +SiR and +SiF showed a significant increase in the SPAD index (Table 1). The SPAD increment during resupply determined by the SPAD index after Fe resupply minus the SPAD index before Fe resupply was higher for both Si additions, especially for +SiF (Table 4). The SPAD increment of the plants without Fe deprivation (Fe sufficiency) was also calculated by the SPAD values at the end of the experiment minus the SPAD values 5 days before the end of the experiment) and showed that this increment was remarkably lower for the +SiR plants. Moreover, when only the Fe uptake during the resupply was considered by using ^57^Fe as a tracer (Table 4), an increase in Fe in the leaves of the +SiF plants was observed. This fact was also seen when considering the total Fe concentration in the old leaves (Table 2). No differences were shown in the Fe/Mn ratio (Table 2). The ROS concentration of the plants treated with a root application of Si presented a significant decrease when compared with the +SiF and –Si plants (Figure 1). No differences were observed in the total phenolic compounds (Table 3), dry weight (Table 1), or MDA concentration between the treatments (Table 3). No differences were observed on the translocation rate of Fe from the root to the shoot (data not shown).

With respect to the hormone concentration (Table 5), no significant differences were obtained for IAA between either the +SiR and the -Si treatments. However, the ABA concentration in the shoots, the JA and JA-Ille in the roots and the new leaves, and the CK concentration in the roots increased in the +SiR treatment. However, the SA in the roots, the JA in the old leaves, and the JA-Ille concentration in the old leaves decreased in the +SiR treatment. The foliar addition of Si significantly increased the IAA and ABA concentration in the roots, but no differences in the shoot were observed. The SA and Ck concentration only demonstrated clear differences in the old leaves of +SiF plants, where the SA decreased, and the Cks increased in value. No consistent results for JA were observed, but the JA-Ille value was reduced in all the plant organs tested.

## Discussion

### Effect of Si Addition on Fe-Sufficient Cucumber Plants

The addition of silicon to cucumber plants under optimal nutritional conditions exerted different effects depending on the type of application. When Si was added to the roots (+SiR), a decrease in the SPAD index (Table 1), shoot Fe concentration (Table 2), and Fe/Mn ratio (Table 2) was observed, creating what could be called a “Si mediated Fe deficiency.” As in every biotic and abiotic stress, an increase in the accumulation of ROS had been expected; but this was not observed (Figure 1); however, an accumulation of phenolic compounds in the +SiR plants was recorded (Table 3). Living cells have very efficient antioxidant systems, including enzymatic and non-enzymatic mechanisms to put the ROS under precise control (Foyer and Noctor, 2005). Concerning the non-enzymatic defensive antioxidant responses, the accumulation of phenolic compounds is one of the most important. It has been also reported that the exogenous addition of Si could improve the ability to eliminate the ROS by regulating the enzymatic activity of antioxidants (Torabi et al., 2015; Kim et al., 2016; Tripathi et al., 2017), but this was not analyzed in this paper due to the complexity of the experimental design required to test this. All these aspects reinforced the hypothesis that +SiR could contribute to the reduction in iron uptake in the cucumber plant under Fe sufficiency conditions, promoting the onset of Fe deficiency mechanisms to mitigate the Fe shortage and the toxic effect of ROS. When Si was sprayed onto the leaves (+SiF), the decrease in the SPAD index was not observed (Table 1); the Fe concentration was not significantly altered, nor was the Fe/Mn ratio (Table 2). The ROS (Figure 1) concentration and total phenols (Table 3) remained similar to that observed in the untreated plants. Consequently, no Fe deficiency was provoked when Si was sprayed onto the leaves of the plants. It was necessary to note here that foliar Si addition tested was at the optimal levels reported in previous works (for example Pilon et al., 2013; Peris-Felipo et al., 2020), for different crops. Therefore, the Si amount added through root and leaf applications was not the same, due to the instability of the Si solutions at the high concentrations required to spray shoots if a similar amount of Si should be added through roots and shoots. The MDA concentration (Table 3) indicated that no cell damage was detected under either +SiR or +SiF treatment, probably due to the activation of cell responses to mitigate the Fe deficiency symptoms caused by the addition of +SiR and also to the softness of the damage. Finally, no differences in the dry weight (DW) were found due to the Si treatment used (Table 1). These results were consistent with those obtained in experiments using wheat by Pilon et al. (2013), who observed that the Si application did not show any increase or decrease in the plant biomass at the stage of Fe sufficiency.

Studies by Becker et al. (2020) also suggest that Si supply induced Fe deficiency in rice (a strategy II plant with respect to Fe deficiency) when applied to the roots under Fe sufficiency levels. These authors tested two different iron-chelating agents (EDTA, EDDHA) and an inorganic Fe form (FeSO_4_) as the source of Fe, and in the case of FeEDDHA, three Fe concentrations: low Fe (3.58 μM), optimal Fe (35.81 μM), and high Fe (179.05 μM). The silicon concentrations were applied as 0.1 mM and 1.0 mM silicic acid. They concluded that Si addition to the roots decreased Fe uptake at optimal and high levels of Fe in the solution. They hypothesized that the Si-enhanced Casparian Band (CB) development took place in the exodermis (Fleck et al., 2011; Hinrichs et al., 2017) and therefore reduced the entrance of Fe into the root apoplast. The CB is observed in all higher plants; it comprises depositions of suberin and lignin in the anticlinal cell walls of the endodermis (Ma and Peterson, 2003; Schreiber et al., 2005). It has been suggested that Si(OH)_4_ within the cell walls forms cross-links with phenols that increase the Casparian Band development (Coskun et al., 2019). The exodermal CBs are described as a diffusion barrier that reduces the ion flux into the cortex (Gierth et al., 1999; Gong et al., 2006) and is involved in controlled substance exchange (Kamula et al., 1994). Becker et al. (2020) observed that the apoplastic Fe concentrations significantly increased with an increasing Fe supply but were reduced by a Si supply at the two higher Fe supply levels tested. Moreover, the Si-mediated Fe deficiency promotes the upregulation of the Fe-homeostasis-related genes in the roots (Becker et al., 2020), but the activation of the Fe deficiency strategy was not sufficient to achieve the same Fe concentration in plants with or without Si addition to the roots (Table 2). Carrasco-Gil et al. (2018) also observed that under conditions of Fe sufficiency in rice, a Si supply to the nutrient solution decreased the Fe concentration in shoots, and they concluded that Si increased Fe root plaque formation, which reduced Fe uptake, promoted ROS production, and consequently activated Fe acquisition strategies, as well as increasing the Fe translocation rate to the aerial parts. In any case, both hypotheses are in agreement with a barrier formation related to the addition of Si, which reduces Fe absorption in rice plants under optimal nutritional conditions. In the cucumber experiment performed in this work, Carrasco-Gil et al. (2018) hypothesis should be ruled out because Fe was added with a strong chelating agent, HBED, which prevented iron plaque formation in the roots, but the hypothesis of Becker et al. (2020) could be valid and might explain the results observed in this experiment. Nevertheless, the effect of Si on the Casparian Band of cucumber cells needs to be tested further. Pavlovic et al. (2013) did not observe a decrease in Fe uptake in cucumber plants when Si was applied to the nutrient solution under Fe sufficiency conditions. They grew cucumber plants of the variety “Chinese long” in hydroponics precultured for 7 days with 1 μM Fe and then for a further 7 days under Fe sufficiency (50 μM FeEDTA), with or without Si 1.5 mM as silicic acid. Because only a low Fe concentration (1 μM) was added to the plants after the germination phase, their study could be considered as a Fe resupply experiment and will thus be discussed in the corresponding section of this paper.

Many abiotic stress responses are coordinated by complex signaling networks, involving both phytohormones and ROS (Bartoli et al., 2013; Baxter et al., 2014), and both also play a direct role in controlling cell division activity, thus influencing both the division cycle and cell death (Gutierrez, 2009; Gill and Tuteja, 2010). As far as we know, this is the first time that Fe nutritional status, ROS production, phytohormone regulation, and the cell cycle have been studied in the same set of experiments. This factor will contribute to a better understanding of the mode of action of Si when applied to the roots or the leaves of cucumber crops, under either optimal, deficiency, or resupply Fe conditions.

Cell proliferation relies on the mitotic cycle that includes DNA replication and cell division, to obtain two identical cells. Cell expansion is generally correlated with nuclei size; therefore, if the nucleus is not big enough, the cell will stop its expansion. After chromosome replication is completed, the cells can inhibit their transition to mitosis and change the cell division cycle to the endoreplication cycle, i.e., successive rounds of full-genome replication without mitosis, that leads to an exponential increase in the genome ploidy level (from 2C to 4C, 8C, 16C, and so forth). Ploidy levels are the number of complete sets of chromosomes in a cell. In the 2C level, there are two copies, 4C four copies, and so on. This endoreplication cycle produces polyploid cells, which are very common in plants, and often associated with cell differentiation and an increase in cell size (Melaragno et al., 1993; Traas et al., 1998). In cucumber plants fertilized with Si under Fe sufficiency, the cellular cycle did not show any significant differences in ploidy levels 1C, 2C, 4C, and 8C (data not shown) after the treatment. However, ploidy levels 16C and 32C (Figure 2) significantly increased after +SiR application compared with the +SiF and –Si treatments, showing a lower grade of cellular division and therefore a greater cellular size in the +SiR treatment. There are several hypotheses regarding the role of endoreplication. It has been frequently correlated with an increase in the cell size and an increase in dry weight (Melaragno et al., 1993; Traas et al., 1998), but other authors have questioned this (Kondorosi et al., 2000; Beemster et al., 2002). In our data, no significant increase in the dry weight due to Si application (Table 1) was observed, and so no relationship between endoreplication and DW could be established. Another hypothesis has concluded that an increase in ploidy may confer upon the plant an increased ability to cope with the accumulation of mutations, to alterations in its DNA repair potential, or in its response to DNA damage (Ramírez-Parra and Gutiérrez, 2007). Therefore, the effects of external factors, i.e., an Fe shortage causing stress to the plant, could be minimized by ploidy-mediated cell processes, which would contribute to the so-called memory effect of the plants that can prepare them for future stresses. For example, under mild drought stress, the leaf area of *A. thaliana* can be maintained by expansion, showing increased endoreduplication (Cookson et al., 2006). In the roots of tolerant varieties of Sorghum bicolor, endoreduplication occurred in response to NaCl exposure, while in the non-tolerant varieties, it did not (Ceccarelli et al., 2006). These authors suggest that endoreduplication could be an evolutionary mechanism for tolerating unfit soil conditions. Moreover, metal toxicity seems to affect ploidy as proved by Fusconi et al. (2006) who showed higher levels of ploidy in *Pisum sativum* roots exposed to Cd. The Fe deficiency caused by the addition of Si to the roots of cucumber plants in the present experiments (Figure 2) could also be the cause of the higher ploidy observed in these treatments in comparison to the healthy foliar and Si untreated plants.

To complete the cycle, the hormone concentration has been assessed to determine whether Si addition altered the hormone balance in the cucumber plants. In the +SiR treatment, the auxin, indoleacetic acid (IAA) (Table 5) significantly increased in the roots and the new leaves. Bacaicoa et al. (2009) determined that Fe starvation was associated with significant increases in the IAA concentration in the roots and, mainly, in shoots when compared with the plants receiving Fe. This fact has reinforced the idea that Si causes an Fe shortage in cucumber, even under optimal Fe nutrition when applied to the roots. Schmidt et al. (2000) and Bacaicoa et al. (2009, 2011) have also observed that IAA plays a role, at least at the transcriptional level, in the regulation of some of the Fe deficiency root responses and could also participate in the regulation of the morphological changes in the root related to Fe deficiency (Romera et al., 2006). These responses included cell division at both the embryonic and vegetative stages (Leyser, 2002; Friml, 2003). Therefore, it is possible that the increase in 16C and 32C (Figure 2) in our ploidy study could be a consequence of the increase in IAA concentration found in the new leaves of +SiR plants. The changes in root morphology as a result of the roles played by IAA and Fe deficiency warrants further research in the future. No differences in the old leaves were detected as Fe chlorosis is normally observed in the new leaves. For the +SiF treatment (Table 5), an increase in this hormone in roots was also observed, but in the new leaves, the IAA concentration decreased significantly when compared with the –Si plants. Jasmonic acid is a well-known signal molecule that is implicated in plant defense responses, as well as in H_2_O_2_ accumulation in plant cell cultures (Wang and Wu, 2013) and the inhibition of cell cycle progression (Świątek et al., 2002). Different authors have observed that Si addition to plants subjected to salinity stress significantly decreased JA production, and this was correlated with the lower level of stress showed by these plants (Liang et al., 2005; Epstein, 2009). No differences were obtained for jasmonic acid at +SiR conditions, although a lower JA concentration was obtained for +SiF plants when compared to –Si and +SiR. However, the beneficial effect of decreasing JA concentration was not recorded in the +SiF plants. The analysis of abscisic acid, cytokinins, and salicylic acid (Table 5) did not provide any consistent results that may have enabled +SiR and +SiF treatment differentiation. However, the CK concentration in the new leaves of plants with both Si additions was higher than in –Si plants. Markovich et al. (2017) also observed a similar increase in cytokinin biosynthesis with silicon exposure when monitoring the dark-induced senescence of sorghum and Arabidopsis plants. So this effect is independent of the method of Si application and the plant species studied.

In summary, Si root supply induced Fe deficiency in well-nourished cucumber plants and the onset of the homeostasis response, the production of phenolic compounds that detoxify ROS high production, due to stress. ROS production activates the hormone responses related to the Fe starvation and influences the cell cycle, promoting endoreplication that is supposed to prepare the cell to cope with future stresses. This fact will be revised in the following sections when the resupply experiment is discussed. Conversely, Si application to the leaves did not alter the Fe nutritional status of the plants, and the decrease in the JA concentration could be indicative of a beneficial effect due to this application.

### Effect of Si Addition on Fe-Deficient Cucumber Plants

Under Fe deficiency (−Fe), a significant decrease in the Fe concentration and the Fe/Mn ratio in the shoot was observed in the +SiR plants compared with –Si (Table 2). However, there were no significant differences in the dry weight and the SPAD index between plants grown with or without Si (Table 1). This was probably due to the activation of the Strategy I response or to the plant’s entrance into the endoreduplication cycle during the Fe sufficiency preculture, to cope with the larger Fe shortage in +SiR treatment. These data differed greatly from those documented by Pavlovic et al. (2013) and Bityutskii et al. (2014), who observed a significant increase in the root and shoot dry biomass, the SPAD index, and the leaf Fe concentration of the Fe-deficient cucumber plants when 1.5 mM Si(OH)_4_ was applied to the nutrient solution, after a 7- or 14-day Fe deficiency (Pavlovic et al., 2013; Bityutskii et al., 2014); however, these effects were not observed after a 21-day growth (Gonzalo et al., 2013). It has been suggested that this fact was due to the previous low Fe application (1 μM) during 1-week seedling preculture (Pavlovic et al., 2013) or to the direct application of –Fe conditions without a Fe preculture (Bityutskii et al., 2014) by comparison with 2-week preculture with 20 μM Fe (Gonzalo et al., 2013) and 1 week in the present research work. Pavlovic et al. (2013) and Bityutskii et al. (2014) also concluded that the Si amelioration of Fe deficiency symptoms involved an increase in Fe in the apoplastic root pools and of Fe transport from root to shoot due to the Si-mediated biosynthesis of Fe chelating compounds. On the contrary, Gonzalo et al. (2013) observed that total Fe content in the whole plant was lower when Si was applied to the nutrient solution. In studies using rice, the decrease in Fe concentration due to the application of Si to roots was not well established, as Becker et al. (2020) did not find any significant differences, although studies carried out by Carrasco-Gil et al. (2018) did. Moreover, Carrasco-Gil et al. (2018) did not detect any increase in the apoplastic Fe with Si addition. The data obtained by Carrasco-Gil et al. (2018) and Becker et al. (2020) using rice, as well as Gonzalo et al. (2013), together with the data presented in this paper supports the hypothesis that Si causes apoplastic obstruction (Coskun et al., 2019). Coskun’s model indicated that the Si(OH)_4_ effect was performed only by the physical deposition of this element in the plant cell but that it did not promote any direct biochemical or metabolic changes, which is in agreement with the lack of specificity of Si transporters (Coskun et al., 2019).

ROS concentration (Figure 1) and total phenolic compound concentration (Table 3) of +SiR were significantly higher than in the -Si plants. In contrast with the data observed under Fe sufficiency, phenolic compound accumulation in the +SiR plants was not sufficient to mitigate the high ROS concentration in +SiR (Figure 1); therefore, in this situation the ROS concentration in the +SiR plants was higher than in the –Si. In addition to acting as antioxidants, the phenolics also have another role described by Jin et al. (2007) “as promotors of the root Fe pools” remobilization in red clover (Jin et al., 2007). In this experiment, the Fe translocation rate from root to shoot did not show any significant differences between the treatments (data not shown). Cell damage (MDA concentration, Table 3) was also higher in the +SiR plants compared with that in –Si. When comparing treatments using a foliar spray (+SiF), no differences were found in the Fe concentration, the ROS, the phenolic compounds, or MDA concentration when compared with the –Si plants, and only the Fe/Mn ratio in the new leaves showed a significant decrease due to this Si application.

Similar to the results of treatments under conditions of Fe sufficiency, hormonal responses under Fe deficiency (Table 5) showed a significant increase in the IAA concentration in the root and new leaves of the +SiR plants when compared with the -Si plants. This finding supports the idea of an induced extra Fe deficiency generated by the addition of Si to the roots (Bacaicoa et al., 2009). Likewise, in this experiment the ABA concentration resulted in a large increase in the number of new leaves on the +SiR plants, but no differences were observed in the roots or the old leaves when compared with the –Si plants. The new leaves were grown without Fe in the nutrient solution and, as expected, under Fe starvation hormonal changes occurred mainly in this portion of the shoot. These results were in accordance with the observations of Jia et al. (2002) relating to an increase in ABA concentration in abiotically stressed plants but differed from those for the specific Fe deficiency stress in cucumber, which showed a decrease in this plant’s regulator in the roots under an Fe shortage. Although this decrease was transient and only expressed at the beginning of the Fe deficiency (Bacaicoa et al., 2009), the length of the experiment will affect the results depending on how long after the samples were taken. In the shoot, the effect was less clear. By analogy with the classic systemic acquired resistance (SAR) response (a long-distance signaling response following exposure to pathogens), a systemic acquired acclimation (SAA) is activated after the plant’s perception of abiotic stress (Xia et al., 2015). Nowadays, hormones such as ABA are considered to be intermediates of the long-distance signaling response to several stresses such as nutrient deficiency (Forde, 2002). As with SA- and JA-mediated systemic signaling, ABA accumulation might regulate the cell-to-cell propagation of ROS and amplify the propagation of the SAA responses. The relationship between ROS and plant hormones, such as JA, ABA, and SA, may explain the mechanism that regulates the systemic signaling responses. Some studies carried out on tomatoes concluded that ABA could induce ROS accumulation in the chloroplasts (Zhou et al., 2014), which is in agreement with the ROS concentration increment measured in the +SiR plants in the present experiment (Figure 1), but does not support the +SiR favored Fe deficiency recovery in this crop, as several authors have hypothesized, i.e., Pavlovic et al. (2013) and Bityutskii et al. (2014). Finally, because ABA inhibits cell division, the cells devoted to the endoreplication cycle increased, which is in perfect agreement with the data presented in Figure 2 and will contribute to the memory effect on these plants. These findings illustrate the complexity of ROS/ABA/cell cycle interactions, particularly under stress, and illustrates that Si addition did not benefit the Fe stress mitigation but probably made the plants more prepared in the event of further stress in the future, through cell switch into the endoreplication cycle. The role of salicylic acid (SA) in the tolerance of the abiotic stress has been studied, the results showed the levels of endogenous SA and ROS that increased in stressed plant cells, and the absence of SA can suppress the production of antioxidant enzymes (APX and CAT) which are responsible for ROS degradation (Hossain et al., 2015). Interestingly, it has been observed that Si supply significantly decreased SA regulation in metal toxicity conditions (Kim et al., 2015). Vlot et al. (2009) proposed that SA and ROS, mainly H_2_O_2_, formed a self-amplifying feedback loop in response to both abiotic and biotic stress conditions. Our data did not confirm the action of Si in the Fe shortage signaling, because the SA concentration (Table 5) increased in the new leaves of the +SiR plants compared with the –Si plants. This fact is in agreement with the increase in ROS in this treatment (Figure 1) but did not correspond with the conclusions of Kim et al. (2015) regarding the Si reduction of SA levels under metal toxicity. In the root and the old leaves, the SA concentration decreased in +SiR compared with the plants without Si addition. Further research on metal deficiencies and SA interactions in different plants organs is required. Several authors have carried out studies into different crops exposed to heavy metal toxicity or salinity stress and have concluded that Si application to the roots reduced JA levels (Liang et al., 2005; Epstein, 2009; Kim et al., 2014), and this was correlated with a lower level of stress induced by Si. JA concentration significantly increased in the roots and the new leaves of +SiR plantlets, which as expected did not contribute to the mitigation of Fe deficiency symptoms (Table 1) due to Si supply. These data confirm our hypothesis that the addition of Si to cucumber roots promotes Fe deficiency and indicates that Si does not mitigate Fe deficiency in this crop, but the behavior of several hormones, i.e., CKs, suggests that cell division could be affected by Si addition, and this prepares plants to cope with future stresses. Furthermore, Bacaicoa et al. (2009) observed that Fe deficiency reduced the concentration of several cytokinins (CKs) in the roots and the shoots. Similar findings were obtained for CKs in the roots of the +SiR plants. However, in the old and the new leaves a significant increase in the +SiR was found. This could be related to a greater biosynthesis of cytokinins due to the external application of Si (Markovich et al. (2017). Cytokinins have been known for a long time to induce cell division (Mok, 1994), which will reduce the number of plants that go through the endocycle; therefore, the lower percentage of events found for –Fe compared with +Fe conditions (Figure 2) could be related to an increase in CKs.

Silicon supply to the leaves (+SiF) showed a significant decrease in IAA and JA and an increase in the SA concentration in the new leaves (Table 5) compared to the –Si plants, which means a beneficial effect on Fe chlorosis compared with the untreated plants. However, according to the SPAD index, DW (Table 1), Fe concentration (Table 2) and ROS data (Figure 1), no differences were observed between these two treatments.

In summary, Fe deficiency increased ROS concentration in the cells, and this acted as a signal amplified by plant hormones [IAA Bacaicoa et al. (2011), SA and ABA (Forde, 2002)] and moved from shoot to root, with the final activation of the Fe-stress physiological root responses. The +SiR treatment promoted an extra Fe shortage in Fe-deficient cucumber plants. However, the Fe deficiency symptoms of +SiR were similar to the Si plants, probably due to the activation of the Strategy I response, or to their entrance into the endoreduplication cycle during the Fe sufficiency precultures. Conversely, +SiF did not significantly augment the Fe deficiency status of the plants, but at the hormonal level a slight beneficial effect was observed.

### Effect of Si Addition After Fe Resupply of Cucumber Plants

This is to the best of our knowledge the first time in which the Si effect on Fe resupply has been specifically tested. As described above, Si applied to the roots significantly increased the number of cells under the endoreplication cycle, and so it was to be expected that differences in the behavior of these plants were detected. Furthermore, the extra activation of Strategy I to cope with Fe deficiency, under both Fe sufficiency and Fe deficiency, when Si was added to the nutrient solution, should improve the plant’s response when it was resupplied with Fe. Moreover, to differentiate Fe addition before and after the resupply, an ^57^Fe stable isotope was used.

During the Fe resupply (−Fe + Fe), the plants that had been treated either with +SiR and +SiF showed a significant increase in the SPAD index with respect to -Si (Table 1). Therefore, the addition of Si (+SiR and +SiF) significantly favored the recovery from the symptoms of Fe chlorosis. Similarly, the SPAD increment during resupply determined by the SPAD index after Fe resupply minus the SPAD index before Fe resupply was higher for both Si additions, especially for +SiF. The SPAD increment of the plants without Fe deprivation (Fe sufficiency) was also calculated by the SPAD values at the end of the experiment minus the SPAD values 5 days before the end of the experiment. Surprisingly these plants also showed a remarkably lower value for the +SiR plants (Table 4), indicating the key role played by Si on stress situations, but not under well-nourished conditions, in which its effect is almost negative. No specific literature was found regarding the process by which Si favored the recovery of iron chlorosis after Fe re-fertilization; however, the results obtained here agree with the beneficial effects of Si demonstrated by several authors. For example, Silva et al. (2012) confirmed their hypothesis about the beneficial effect of Si by increasing the amount of chlorophyll in tomato plants under water stress conditions when Si was applied via the roots. Furthermore, if we consider the work carried out by Pavlovic et al. (2013), and Bityutskii et al. (2014), into Fe resupply experiments due to the lack, or quasi lack, of Fe during of the preculture of the plants, this effect was also observed by these authors using cucumber. Moreover, only when the Fe uptake during the resupply was examined using ^57^Fe as a tracer (Table 4) were increased levels of Fe in the leaves observed related to +SiF treatment, which is in agreement with the total Fe concentration (Table 2) for the old leaves of the +SiF plants. Interestingly, the ROS concentration of the +SiR plants showed a significant decrease when compared with the +SiF and –Si plants (Figure 1), which was the opposite of their behavior in the Fe deficiency conditions but similar to the Fe sufficient conditions. Here, phenolic compound production was not in accordance with a decreased ROS in the +SiR plants (Table 3), but the low ROS concentration in these plants could be related to the ploidy enhancement (Figure 2), which provides better resources for the plants to cope with stresses and to recover from them.

With respect to the hormone concentration (Table 5), no significant differences in IAA were obtained between the +SiR and -Si treatments, indicating that plant recovery from Fe chlorosis was similar for both treatments. However, the higher ABA concentration in the shoots, as well as an increase in JA and JA-Ille in the roots and new leaves of +SiR, when compared with –Si, –indicated that these plants had been subjected to stress. These results are in agreement with the higher MDA values obtained for +SiR treatment (Table 3). Conversely, the low levels of SA in the +SiR roots compared with the –Si plants, might indicate an effective Fe deficiency recovery in the +SiR treatment because it has been demonstrated that under abiotic stress, the SA levels increase in plant cells (Hossain et al., 2015). Alternatively, work carried out by Kim et al. (2015) has demonstrated that Si significantly decreased the SA hormone concentration, which may explain the low concentration of SA observed here. The foliar addition of Si (+SiF) did not show any differences in IAA, SA, CKs, and ABA concentration in the new leaves when compared to –Si. No consistent results for JA were observed, but the JA-Ille value was reduced in all the plant organs tested. Further research with different resupply periods is necessary to obtain consistent conclusions regarding the hormone response at re-fertilization.

In summary, under the Fe resupply conditions, the addition of Si either by foliar or radicular application increased the SPAD index (Table 1). Furthermore, the SPAD index increment, both before and after resupply, was higher for the +SiR plants than for the +SiF plants, which was probably related to the longer period of Fe deficiency (under + Fe and –Fe) of the +SiR plants. However, only the foliar application of Si increased the Fe in leaves with respect to the –Si control plants. Finally, the ROS concentration in the +SiR plants was reduced with respect to the +SiF and –Si treatments, indicating that these plants had recovered from the chlorosis faster than the others, which was possibly related to an increase in the endoreplication cycle, which prevents plants from damage and may confer on them a greater capacity to respond to stresses.

## Conclusion

The addition of silicon to the roots of cucumber plants induced Fe deficiency in plants grown under optimal Fe nutritional conditions. Furthermore, under Fe deficiency conditions, the Si root supply induced an additional Fe shortage. A foliar application of silicon to the leaves did not induce Fe shortage in the plants. Plant recovery after Fe resupply was more effective in the Si-treated plants than in the untreated plants: the foliar treatment resulted in an increase in the Fe concentration in the leaves, and the addition of Si to the roots significantly reduced plant stress indicators, such as ROS concentration. These results indicate a quicker recovery from the chlorosis, which was possibly related to an increase in the endoreplication cycle which causes the plant memory effect. Si fertilization, especially when sprayed to the leaves, could be used as a preventive tool to minimize Fe chlorosis symptoms.

The mode of action of silicon appeared to be more related to the induction of the Fe deficiency responses, than to any direct biochemical or metabolic involvement of this beneficial element on the signaling pathway that triggers ROS hormones and cell cycle changes. However, the effects of Si on hormone levels such as CKs or SA could only be explained by Si addition. Therefore, although the physical effect of Si in cucumber plants has been reinforced, its biochemical or metabolic role could not be completely ruled out.

## Data Availability Statement

All datasets generated for this study are included in the article/supplementary material, further inquiries can be directed to the corresponding author.

## Author Contributions

SC-G and LH-A: conceptualization and methodology. SC-G, CC, LE, JG-M, and ÁZ: formal analysis. SC-G, CC, and LE: investigation. LH-A: writing—original draft and funding acquisition. SC-G, LH-A, and JG-M: validation and writing—review and editing. All authors contributed to the article and approved the submitted version.

## Conflict of Interest

The authors declare that the research was conducted in the absence of any commercial or financial relationships that could be construed as a potential conflict of interest.
